# Frequency and factors associated with carriage of multi-drug resistant commensal *Escherichia coli* among women attending antenatal clinics in Central India

**DOI:** 10.1186/1471-2334-13-199

**Published:** 2013-05-02

**Authors:** Ashish Pathak, Salesh P Chandran, Kalpana Mahadik, Ragini Macaden, Cecilia Stålsby Lundborg

**Affiliations:** 1 Global Health (IHCAR), Department of Public Health Sciences Karolinska Institutet, Tomtebodavägen 18 A 9, Stockholm 17177, Sweden; 2 Department of Pediatrics, R.D. Gardi Medical College, Surasa, Ujjain 456010, India; 3 Department of Women and Children’s Health, International Maternal and Child Health Unit, Uppsala University, Uppsala, Sweden; 4 Department of Microbiology, St Johns Research Institute, Bangalore, India; 5 Department of Obstetrics and Gynaecology, R.D. Gardi Medical College, Ujjain 456010, India

## Abstract

**Background:**

Commensal *Escherichia coli* are a prominent reservoir of genes coding for antibiotic resistance and also responsible for endogenous infections in pregnant women. We studied the factors in pregnant women associated with carriage of multi-drug resistant (MDR) *E. coli* and genetic determinants of antibiotic resistance in them.

**Methods:**

Women attending to Obstetric and Gynaecology department outpatient clinics for routine antenatal check-up were administered a questionnaire. Peri-anal swabs were collected for culture isolation and identification of *E.coil.* Antibiotic sensitivity was done using the Kirby-Bauer disc diffusion method as recommended by the CLSI guidelines. MICs for quinolones and third generation cephalosporins were done using the agar dilution method. Genes coding for production of beta lactamses and for the quinolone resistance determinant were screened by polymerase chain reaction. Rep-PCR was done on MDR isolates for detecting possible genetic similarity. Multiple logistic regression models were used to determine the independent factors associated with carriage of MDR isolates.

**Results:**

A total of 710 isolates of *E. coli* from 710 women (mean age 26 years) were included in the study. Resistance to at least one antibiotic tested was detected in 94% of the *E. coli* isolates. A total of 109 isolates were ESBL producing and 35 isolates were MDR. In the MDR isolates MIC_50_ and MIC_90_ for quinolones and third generation cephalosporins were high for those isolates that carried *bla*_TEM_ gene (26 isolates) and *bla*_CTX-M_ gene (24 isolates). Both *bla*_TEM_ and *bla*_CTX-M_ genes were detected in 19 isolates. The commonest Plasmid Mediated Quinolone Resistance (PMQR) gene identified was *aac(6*^*′*^*)-Ib-cr* (n = 23/25). All isolates carrying the PMQR genes were also positive for *bla*_CTX-M_ and *bla*_TEM_ gene. Mutations in *gyr A* and *par C* genes were present in all 35 MDR isolates. The statistically significant risk factors for carriage of MDR *E. coli* were graduate or post-graduate education, a self-employed status, a family size of more than 10 members, antibiotic usage in last four weeks, and history of hospitalization in the last four weeks.

**Conclusions:**

The presence of genes coding for extended spectrum of beta lactamases and plasmid mediated quinolone resistance in commensal *E. coli* is disconcerting. The study provides strong basis good antibiotic stewardship.

## Background

The worldwide dissemination of plasmid-borne extended-spectrum beta-lactamases (ESBLs) in *Enterobacteriaceae* has become a major global public health problem [1]. Inappropriate use of antibiotic is the predominant selection pressure for antibiotic resistance and aids horizontal transfer of bacterial resistance [2]. The spread of multi-resistant strains of bacteria in the community is compounded with a paucity of new classes of antibiotics effective against Gram-negative bacteria like *Escherichia coli*[3]. *E. coli* is the most common bacterial cause of urinary tract infections (UTI’s) in women and also a major cause of catheter induced UTI, surgical site infections, intra-abdominal infections and sepsis in all age groups and meningitis in neonates [4]. The recent spread of New Delhi metallo-beta-lactamase-1 (NDM-1) producing bacteria emphasises the fact that resistant bacteria respect no geographical boundaries [5].

*E. coli* forms part of the bacteria commensal flora of the human gut. It has been identified as the predominant reservoir of antibiotic resistance genes [2]. Once acquired, these resistance genes are stable and are easily transferable to pathogenic bacteria [6]. The published studies on antibiotic resistance in commensal *E. coli* in resource-poor settings are limited as resistance monitoring is resource intensive, requires dedicated staff and a well-equipped laboratory [7-9].

During pregnancy many anatomical and physiological changes occur, which also affect the urinary tract. Asymptomatic bacteriuria is reported to occur in 2–10% of pregnancies and *E. coli* causes 70–80% of them [10]. Most infections are endogenous in origin [10]. Asymptomatic bacteriuria can have serious consequences for the foetus and/or mother [10]. However, knowledge about the risk factors for carriage of resistant *E. coli* in the women of reproductive age group is lacking in general and especially from India. Screening the commensal *E. coli* in antenatal women for antibiotic resistance pattern will provide guidelines for empiric therapy especially for sick new-borns. Molecular analysis of these isolates will provide prevalence of antibiotic resistant genes present in *E. coli* in the community. The aim of the study is to ascertain the factors associated with the carriage of resistant commensal *E. coli* in pregnant women and further to characterize the genotypic determinants of antibiotic resistance in them.

## Methods

### Study setting, study participants, enrolment and survey procedure

The study was conducted in Obstetrics and Gynaecology outpatient clinics of a teaching and a non-teaching hospital in Ujjain, Madhya Pradesh, India during a 15- month period from November 2007 to February 2009. Both the hospitals cater predominantly to rural populations. Women between 18 to 49 years of age coming for routine antenatal check up were included in the study. Patients requiring emergency admission or with genital infections were not included.

Study participants were interviewed using a questionnaire containing details of patient particulars: age, family size, education, occupation of the main breadwinner, a reported history of antibiotic use in the previous four weeks and reported hospitalization in the previous four weeks. The questionnaire was piloted for one month to ascertain feasibility of data collection and to train the study assistants. Verbal informed consent was obtained from all participants after explaining the purpose of the study. The Ethics Committee of R.D. Gardi Medical College approved the study (approval number 41/ 2007).

### Sample collection, isolation and identification of *E. coli*

Perianal swabbing was done using sterile, moistened cotton swabs. The collected samples were placed in Amies transport media with charcoal (HiMedia, Mumbai, India). Swabs were transported on ice to microbiology diagnostic laboratory within four hours of collection. Swabs were plated on MacConkey agar. All swabs grew *E. coli*.

*E. coli* were identified by standard recommonded methods [11]. Five colonies morphologically resembling of *E. coli* were selected and identified biochemically [11]. A single isolate from the pool of five colonies was selected for further characterization.

### Phenotypic determination of antibiotic susceptibility

Antibiotic susceptibility testing was done using the Kirby-Bauer disc diffusion method. Disc strengths were as recommended by Clinical and Laboratory Standards Institute (CLSI) guidelines [12]. The plates were incubated at 35°C for 18 to 24 hours. Diameter and inhibition zones produced by the antibiotic disc were measured to the nearest millimetre. *E. coli* ATCC 25922 was used as the reference strain. Antibiotics tested were: ampicillin, cefuroxime, ceftriaxone, cefixime, ceftazidime, nalidixic acid, norfloxacin, ciprofloxacin, ofloxacin, gentamicin, amikacin, chloramphenicol, tetracycline, co-trimoxazole, nitrofurantoin, amoxicillin/clavulanate and piperacillin/tazobactum and imipenem. For calculations all isolates showing zone of inhibition indicating intermediate susceptibility were considered resistant. Multi-drug resistance (MDR) isolates were defined as those isolates having co-resistance to at least three different antibiotic classes [13]. Resistance of the MDR isolates was confirmed by Minimum Inhibitory Concentration (MIC) using agar dilution method for quinolones (ciprofloxacin, ofloxacin) and third generation cephalosporins (cefotaxim, ceftazidime) according to CLSI guidelines [12].

Extended Spectrum Beta-Latamase (ESBL) were detected phenotypically by the combined disc diffusion method with cefotaxime (30µg) and cefotaxime/clavulanic acid (30/10 µg) and ceftazidime (30µg) and ceftazidime/clavulanic acid (30/10 µg) according to CLSI guidelines [12].

### Detection of genotypic determinants for ESBLs and resistance to quinolones

Multiplex Polymerase Chain reactions (PCR) were used to detect genes coding for beta-lactamases. Total bacterial DNA from selected MDR *E. coli* showing ESBL production phenotypically was extracted by alkaline lysis method [14]. Amplification and identification of ESBL encoding genes (*bla*_*CTX-M*_, *bla*_*TEM*_ and *bla*_*SHV*_) was done using previously described primers [15].

A separate multiplex PCR was done for detecting plasmid mediated quinolone resistance genes (PMQR; *qnrA, qnrB, qnrS, aac(6*^*′*^*)-Ib-cr* and *qepA*) using previously described primers [16]. After PCR amplification, products were visualized under gel documentation system. Chromosomally mediated quinolone resistance was detected using PCR and DNA sequencing of *gyr A* and *par C* genes [17]. The purified PCR products were sequenced with ABI 3730XL (Applied Biosystems, USA) sequencer. The nucleic acid sequences were analyzed by Basic Local Alignment Search Tool available at the National Center for Biotechnology database. The nucleic acid sequences were submitted to Genbank (GenBank accession numbers- KC795246 - KC 795255, KC795239 - KC795245, KC788561, KC788562).

### Rep-PCR typing of MDR *E. coli* isolates

The molecular epidemiology of the MDR *E. coli* was done by Rep-PCR using BOX A1R (5^′^-CTACGGCAAGGCGACGCTGACG-3^′^) primers as previously described [18]. PCR analysis was performed on a total of 25 ml reaction mixture using 3 µl of total bacterial DNA, as previously reported. PCR products were then separated by electrophoresis in 1.5% agarose gels with 1XTAE running buffer at 8V/cm for 70 minutes and stained with Ethidium bromide (0.5µg/ml), (Sigma Aldrich), for 30 minutes and visualized using a UV trans-illuminator. DNA molecular weight marker (O’GenerulerTM 100 bp DNA ladder; Fermentas) was used as a molecular size standard. Gel images were normalized, bands were identified and the data were statistically analysed by using Bionumerics software version 3.5 (Applied Maths, Kortrijk, Belgium). Clustering of patterns was performed by Unweighted Pair Group with Arithmetic averages (UPGMA) and dendrogram was created using the Dice Similarity Coefficient [18].

### Statistical analysis

The data was entered in EpiData Entry (version 3.1) and then transferred to Stata 10.0 (Stata Corp. College Station, Texas, USA) software for statistical analysis. Frequency and percentages are presented for categorical data. Pearson Chi square test with 95% confidence interval (95% CI) was used to calculate odds ratios (OR) for potential risk factors associated with outcome variable carriage of MDR isolates of *E. coli*. Crude ORs were calculated from two by two tables. Multi-variable logistic regression models were used to calculate adjusted OR in the final model. The dichotomized explanatory variables included were, occupation of the breadwinner (self employed versus salaried), reported antibiotic use in the previous four weeks (yes versus no), reported hospitalization in the previous four weeks (yes versus no). Categorical variables included, age group in years (15 to 19, 20 to 29, 30 to 39 and 40 to 45), family size (less than or equal to 4, 5 to 10 and more than 10 members) and education (illiterate, up-to primary, up-to higher secondary and graduate or post-graduate). A *P* value less than or equal to 0.05 was considered significant. The goodness of fit for the model was tested by examining the Receiver Operating Characteristics (ROC) curve plot [19]. The area under the ROC curve was 0.944 for the model for MDR *E. coli* meeting the criteria for excellent model determination [19].

## Results

### Study population and enrolment

A total of 730 women were approached of which 710 were enrolled. Of the 710 women enrolled, 412 (58%) were from teaching hospital and remaining from the non-teaching hospital. The mean age of the women was 26 years (95% confidence interval, CI 26 to 27, range 15 to 44 years) (Table 1).

**Table 1:** **Multiple logistic regression analysis of factors associated with carriage of multi-drug resistant *E. coli *among healthy women attending antenatal clinics in two hospitals in Ujjain, India**

**Factor**	**Total**		**MDR**			
	**n = 710**	**%**^**a**^	**n = 35**	**%**^**a**^	**OR (95% CI)**	**P value**
Age (in years)^b^						
15 to 19	55	95	2	5	1	
20 to 29	434	94	20	6	0.59 (0.13-2.67)	0.49
30 to 39	201	94	10	6	0.50 (0.09-2.70)	0.42
40 to 45	20	80	3	20	2.19 (0.23-20.9)	0.49
Education^b^						
Illiterate	393	94	17	6	1	
Up-to primary	240	97	6	3	0.69 (0.21-2.18)	0.53
Up-to higher secondary	47	91	3	9	1.27 (0.26-6.27)	0.76
Graduate or post graduate	30	60	9	40	**3.98 (1.05-15.10)**	**0.04**
Occupation						
Salaried	207	87	19	13	1	
Self-employed	503	96	16	4	**0.41 (0.15-1.06)**	**0.06**
Family size^b^						
Less than or equal to 4	339	94	14	6	1	
Between 5–10	321	96	10	4	0.51 (0.17-1.47)	0.21
More than 10	50	70	11	30	**4.97 (1.51-16.33)**	**0.008**
Reported antibiotic usage (last 4 weeks)						
No	663	97	13	3	1	
Yes	47	36	22	64	**17.01 (6.35-45.51)**	**<0.001**
Reported Hospitalization (last 4 weeks)						
Yes	659	97	13	3	1	
No	51	41	22	59	**26.99(10.32-70.5)**	**<0.001**

%^a^ row percentage; ^b^ The first value within the variable acted as reference value for logistic regression; OR- Odds ratio; CI-confidence interval;

OR significant at *P* **=** 0.05 are shown in bold face; MDR- multi-drug resistance (co-resistance to three different classes of antibiotics).

### Detection of antibiotic-resistant phenotype and factors associated with carriage of MDR *E. coli*

The antibiotic resistance pattern to selected antibiotics for 710 *E. coli* isolates from 710 antenatal women is shown in Table 2. A high number (n = 668, 94%; 95% CI 92.4 to 95.8) of *E. coli* isolates showed resistance to at least one of the groups of antibiotics tested. All the isolates were susceptible to imipenem. Resistance was found to the commonly prescribed oral antibiotics, nalidixic acid (77%), tetracycline (69%), ampicillin (55%), co-trimoxazole (48%), cefixime (32%), ciprofloxacin (28%) and amoxicillin/clavulanate (27%). The resistance to third generation cephalosporins was seen in the range of 17 to 19% and that to piperacillin/tazobactam and amikacin 3% and 7% respectively.

**Table 2:** **Co-resistance pattern of ten antibiotics for 710 *E. coli *isolates from healthy women attending antenatal clinics in two hospitals, Ujjain, India**

	**n (%)**	**Amoxycillin/Clavn (%)**	**Pipracillin/Tazobactumn (%)**	**Ceftriaxonen (%)**	**Cefiximen (%)**	**Ciprofloxacinn (%)**	**Amikacinn (%)**	**Tetracyclinen (%)**	**Co-trimoxazolen (%)**	**Chloramphenicoln (%)**
Ampicillinn (%)	388 (55)	183	19	127	172	170	32	317	251	34
		(26)	(3)	(18)	(24)	(24)	(5)	(45)	(35)	(5)
Amoxycillin/clav n (%)	189(27)	-	**15**	114	139	127	21	173	163	27
			**(2)**	(16)	(20)	(18)	(3)	(24)	(23)	(4)
Pipracillin/tazobactumn (%)	23		-	**12**	19	**15**	**7**	23	18	21
	(3)			**(2)**	(3)	**(2)**	**(1)**	(3)	(3)	(3)
Ceftriaxonn (%)	133			-	129	107	18	124	116	24
	(19)				(18)	(15)	(3)	(17)	(16)	(3)
Cefiximen (%)	226				-	130	23	117	172	33
	(32)						(3)	(16)	(24)	(5)
Ciprofloxacinn (%)	198					-	25	182	160	26
	(28)						(4)	(26)	(23)	(4)
Amikacinn (%)	47						-	44	25	20
	(7)							(6)	(3)	(3)
Tetracyclinen (%)	491							-	278	39
	(69)								(39)	(5)
Co-trimoxazolen (%)	340								-	31
	(48)									(4)
Chloramphenicoln (%)	40									-
	(6)									

Values in bold show combination of antibiotics for which resistance is less than three percentages.

Of the 127 isolates that were resistant to third generation cephalosporins, 109 isolates were seen to produce ESBLs and of these 35 isolates were MDR. MIC_50_ and MIC_90_ for cefotaxime, ceftazidime, norfloxacin, ciprofloxacin and ofloxacin were calculated for these isolates (Table 3). The assay for MIC showed high levels of resistance to third generation cephalosporins and quinolones.

**Table 3:** **MICs of 35 MDR commensal *E. coli *isolates**

**Antibiotic**	**Range (µg/mL)**	**MIC 50 (µg/mL)**	**MIC90 (µg/mL)**
Ciprofloxacin	0.125-512	128	256
Norfloxacin	0.125-512	512	>512
Ofloxacin	0.125-512	64	128
Cefotaxime	0.125-512	>512	>512
Ceftazidime	0.125-512	32	128

The statistically significant factors associated with carriage of MDR *E. coli* are shown in Table 1 and are as follows: “graduate or post-graduate education” Odds Ratio (OR) 3.98 (95% CI 1.05 to 15.10; P = 0.04); “self employed” OR 0.41 (95% CI 0.15 to 1.06; P = 0.05); “family size more than 10 members” OR 4.97 (95% CI 1.51 to 16.33; P = 0.008); reported “antibiotic usage in last four weeks” OR 17.01 (6.35 to 45.51; P < 0.001) and “history of hospitalization in the last four weeks” OR 26.99 (95% CI 10.32 to 70.50; P < 0.001). The statistically significant factors associated with carriage of ESBL producing isolates are: “antibiotic usage in last four weeks” OR 5.21 (3.12 to 11.28; P < 0.001) and “history of hospitalization in the last four weeks” OR 7.82 (95% CI 4.31 to 10.50; P < 0.001).

### Detection of antibiotic resistance coding genes

**Figure 1:**
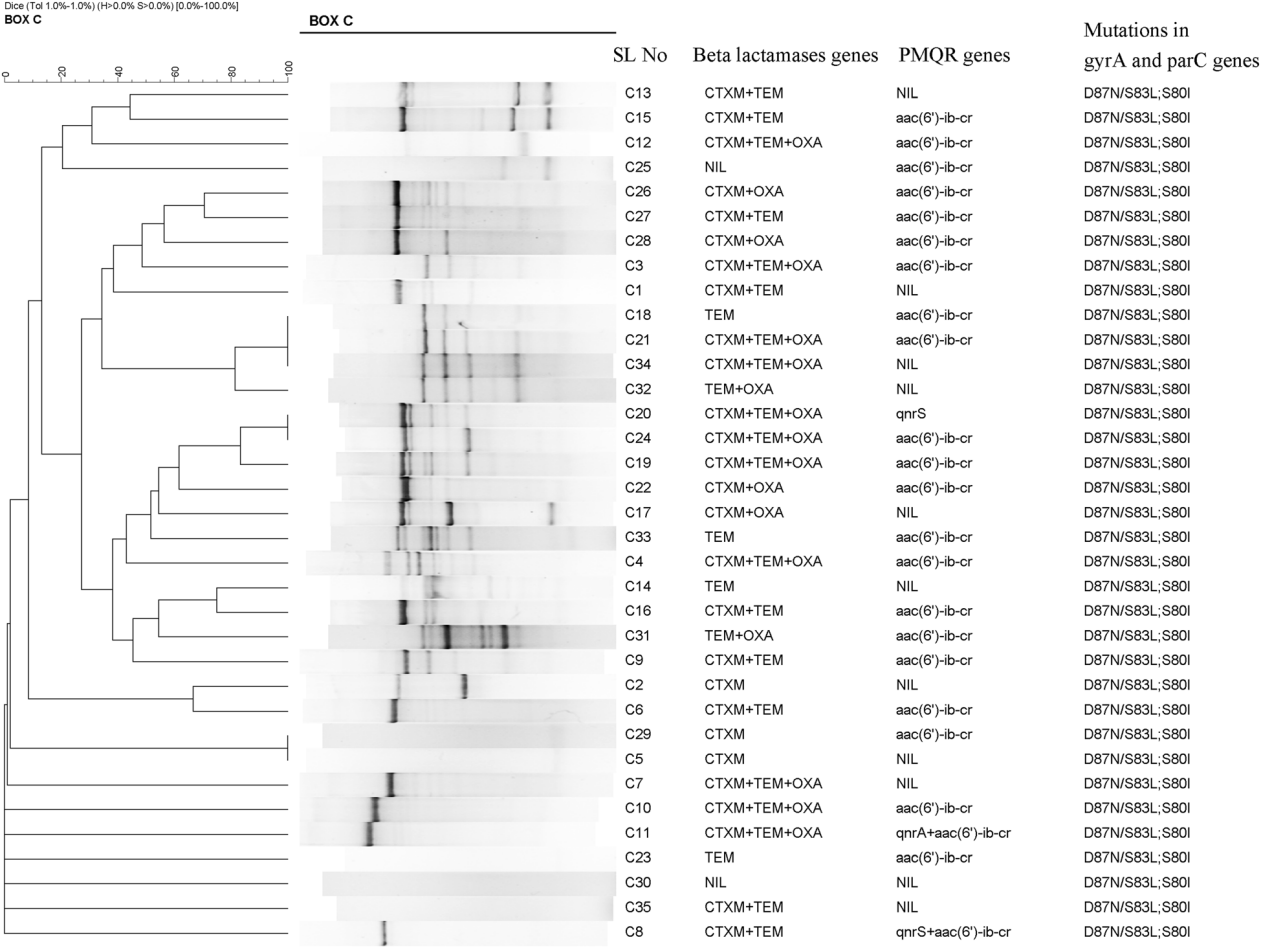
**Dendrogram of Rep-PCR patterns showing the genetic relatedness of 35 MDR *E. coli *isolates.**

The multiplex PCR was done for detection of beta-lactamase producing genes. Of the 35 MDR isolates, plasmid mediated resistance to cephalosporins was due to *bla*_CTX-M-15_ (26/35 = 74%), *bla*_TEM −1_(26/35 = 74%) and *bla*_OXA-1_(17/35 = 49%). The *bla*_CTX-M_-_15_ and *bla*_TEM-1_ were seen in 19 (54%), *bla*_CTX-M-15_ and *bla*_OXA-1_ in 15 (43%), *bla*_TEM-1_ and *bla*_OXA-1_ in 12 (34%) and *bla*_*CTX-M-15*_, *bla*_TEM-1_ and *bla*_OXA-1_ in 12 (34%). All cefotaxime and ceftazidime resistant isolates were also screened for the presence of the *bla*_SHV_ gene and none was found to carry it. We sequenced TEM gene and all were found to be TEM-1. The screening for PMQR genes showed one isolate positive for *qnrA*, one for *qnrS1* gene and 23 isolates were positive for *aac(6*^*′*^*)-Ib-cr*. It was note worthy that all the isolates carrying PMQR genes also carried the *bla*_TEM-1_ and *bla*_CTX-M-15_ gene. There were 17 isolates having both *bla*_CTX-M-15_ and *aac(6*^*′*^*)-Ib-cr* genes (Figure 1). PCR and DNA sequencing for mutations in *gyr A* and *par C* genes indicated chromosomally mediated quinolone resistance. Sequence analysis of these genes indicated that all the 35 isolates had amino acid substitutions at QRDR regions at *gyr A* (D87N/ S83L) and *par C* (S 80l) genes.

Chromosomally mediated resistance to quinolones was seen in all the 35 isolates based on the presence of *gyrA* and *parC* genes. Plasmid mediated resistance to cephalosporins and quinolones was seen in 22/35 (63%). Of these 21 isolates carried the *aac(6*^*′*^*)-Ib-cr* genes and only one isolate carried the *qnrS* along with *bla*_CTX-M-15_, *bla*_TEM-1_ and *bla*_OXA-1_ The other *qnrA* and *qnrS* genes carrying plasmid were seen along with the plasmid coding for cephalosporin resistance. Details of MDR isolates along with MIC values are included in the supplementary data (Additional file 1: Table S1). The dendrogram (Figure 1) depicts the genetic similarity of MDR *E .coli.* The genes coding for plasmid mediated and chromosomally mediated resistance to cephalosporins and quinolones are also depicted at Figure 1.

## Discussion

In the present study it was found that out of the 710 *E. coli* isolated from women attending antenatal clinics in Ujjain, India 94% were resistant to at least one of the antibiotic groups tested. The statistically significant factors associated with carriage of MDR *E. coli* were better education, overcrowding (family size more than 10 members) history of antibiotic use and/or hospitalization in the last four weeks.

Quinolone resistance is usually chromosomally mediated [20]. All 35 multi-resistant isolates showed presence of *gyrA* and *parC* genes, which are chromosomally mediated. The *aac(6*^*′*^*)-Ib-cr* gene accounted for 95% of the PMQR genes. Similar findings in pathogenic *E. coli* have been reported elsewhere in India [21] and in *E. coli* isolated from hospital wastewater in our setting [22]. Plasmid mediated resistance to cephalosporins was largely due to *bla*_CTX-M -15_ which is in keeping with other studies done in India [21,23]. The *bla*_TEM-1_ is not been subtyped therefore no comment can be made for its co-relation with ESBL production. It is interesting to note that *bla*_SHV_ was not detected. The presence of genes coding for extended spectrum of beta lactamases and plasmid mediated quinolone resistance in commensal *E. coli* is disconcerting.

Studies from India [7,24] and other low-middle income countries [25] have reported lower rates of resistance (range of 32 to 63%) to a single antibiotic compared to the present study (94%). The difference in the resistance rates could be due to variation in the geographical area of the study [7,8,26] and also is dependent on the pattern of antibiotic prescribing in the local community [27,28]. The ESBL rate (15%) in the present study is lower than that reported among pathogenic *E. coli* (69%) isolated from patients with clinical infections in the same geographical area [29].

The majorities of women enrolled in the study were either illiterate or had only a primary education (Table 1). Women with higher education had a higher likelihood of carriage of MDR *E. coli.* This could be a chance association and needs further validation. But, women with higher education may also be socio-economically better off and thus more likely to seek health-care and consequently have a greater exposure to antibiotics [30]. Most self-employed women enrolled in the study were daily wage workers, and consequently, probably, had limited opportunity to seek medical care, limiting their antibiotic exposure.

Exposure to hospital environment especially hospitalization in intensive care units is a major risk factor for carriage of multi-drug resistant bacteria especially in resource poor settings where hospitals can have high infection rates and spread of multi-drug resistant pathogens [25,31]. A multitude of factors including poor infrastructure of hospitals, low compliance with hand-hygiene, heavy workload with understaffing, overcrowding, lack of or poorly functioning infection control programme contribute to the problem [31].

In the present study a significantly higher MDR carriage among women living in larger families (more than 10 members), OR 4.97 (95% CI 1.51 to 16.33; P = 0.008) was found. Similar results have been reported in postmenopausal women in our setting [32]. Over-crowding combined with inadequate hygiene and sanitary facilities and sewage disposal, would lead to greater sharing of commensal flora in families and communities [8,32]. In a study among primary school children in rural Tamil Nadu over crowding in classrooms was associated with increased carriage of antibiotic resistant commensal *E. coli*[24]. Thus, the dynamics of bacterial transmission due to over crowding is worthy of further investigation.

The rate and amount of antibiotic use in community an important determinant of increasing antibiotic resistance in bacteria [27,28]. A meta analysis and review on the effect of antibiotic prescribing in a primary-care setting on antimicrobial resistance in individual patients included five studies of urinary tract infections (14348 participants), the pooled odds ratio (OR) for resistance was 2.5 (95% CI 2.1 to 2.9) within two months of antibiotic treatment [33]. The OR at one month (only one study) was 6.1 (95% CI 2.8 to 13.4) [33]. Recent antibiotic use was associated with increased risk of carriage of resistant *E. coli,* OR 1.8 (95% CI 1.5 to 2.3) in a study from Indonesia [25].

The ESBL production is the commonest mechanism of resistance to cephalosporins in *Enterobacteriaceae*[2]. Phenotypic detection of ESBL production was in 15% isolates in the present study, which is much lower than the 51% reported in a study from China [34].

The *bla*_CTX-M-15_ and *bla*_TEM −1_ coded genes are widespread in *E. coli*[2]. The detection of *bla*_CTX-M-15_ and *bla*_TEM-1_ genes as shown in Figure 1 is similar to that in Latin America [9]. In the present study it has been noted that a majority of MDR *E coli* isolates (48%) were carrying *aac(6*^*′*^*)-Ib-cr* gene. These isolates also showed high MIC to ciprofloxacin and ofloxacin probably due to chromosomally mediated resistance (*par A* and *par C*) (Table 3). Co-residence of *bla*_CTX-M-15_, *qnr* and *aac(6*^*′*^*)-Ib-cr* genes on the same plasmid has been reported elsewhere [35].

PMQR genes code for low levels of quinolone resistance [36]. The presence of PMQR gene in MDR isolates was 4%, which is similar to that in a study from Republic of Korea [16]. It is interesting to notice that chromosomal mediated quinolone resistance coded by mutation in QRDR regions of *gyr A* and *par C* genes was present in all MDR *E coli* isolates in the present study. A study in Ghana on quinolone-resistant *E. coli* in the faecal flora also reported the presence of *qnr* genes and amnio acid substitutions at *gyrA* and *par C* genes [37].

An analysis of resistance pattern of commensal *E. coli* can be helpful in deciding empiric treatment of choice for suspected neonatal infections. Neonatal sepsis with multi-drug resistant gram negative organisms resistant to ampicillin, third generation cephalosporins and gentamicin caused high mortality (26%) in neonates in a neonatal intensive care unit (NICU) in India [38]. The current choices of therapy in our NICU are in line with the most sensitive patterns described ie third generation cephalosporins along with an aminoglycoside (Table 2). However, the last resort antibiotics (eg. vancomycin and imipenem) are now increasingly being used in view of increasing resistance [29] in our NICU to salvage septic neonates.

The most important strength of this study is the combination of epidemiological and molecular methods for the identification of factors associated with carriage of resistant bacteria. Antenatal women are a population, which is at a low risk for receiving antibiotics. Despite this a history of antibiotic therapy was identified as an important risk factor associated with carriage of resistant bacteria. The fact underscores the importance of prudent antibiotic use. A structured questionnaire was used which could have resulted in us missing other factors related to carriage of resistant *E. coli* like environmental factors and food chain related factors.

## Conclusions

The presence of antibiotic resistance mediating genes in commensal bacteria provides information on the occurrence of multi-drug resistance bacteria in a given geographical area. The presence of genes coding for extended spectrum of beta lactamases and plasmid mediated quinolone resistance in commensal E. coli is disconcerting.

This is compounded in resource poor settings with overcrowding and poor sanitation, which seem to aid the spread of multi-drug resistant bacteria within a community. This provides strong basis of good antibiotic stewardship.

## Competing interests

The authors declared that they have no competing interests.

## Authors’ contributions

AP, SC, KM, RM and CSL participated in the conception and design of the study. KM and AP collected data in the field. SC and RM carried out the molecular genetic studies, participated in the sequence alignment. AP and SC performed the statistical analysis and drafted the manuscript. CSL coordinated the study. AP, SC, KM, RM and CSL revised the paper critically for substantial intellectual content. All authors read and approved the final manuscript.

## Pre-publication history

The pre-publication history for this paper can be accessed here:

http://www.biomedcentral.com/1471-2334/13/199/prepub

## Supplementary Material

Additional file 1 **Details of resistance mechanisms of the 35 multi-drug resistant *E. coli *among healthy women attending antenatal clinics in two hospitals in Ujjain, India.** Click here for file
